# Emergence of AnnexinV^pos^ CD31^neg^ CD42b^low/neg^ extracellular vesicles in plasma of humans at extreme altitude

**DOI:** 10.1371/journal.pone.0220133

**Published:** 2019-08-01

**Authors:** Olaf Utermöhlen, Kristin Jakobshagen, Birgit Blissenbach, Katja Wiegmann, Tobias Merz, Jacqueline Pichler Hefti, Martin Krönke

**Affiliations:** 1 Institute for Medical Microbiology, Immunology and Hygiene, University Hospital Cologne, Cologne, Germany; 2 Center for Molecular Medicine Cologne (CMMC), Cologne, Germany; 3 Cologne Excellence Cluster on Cellular Stress Responses in Aging-Associated Diseases (CECAD), University of Cologne, Cologne, Germany; 4 Department of Intensive Care Medicine, University Hospital and University of Bern, Bern, Switzerland; 5 Cardiovascular Intensive Care Unit, Auckland City Hospital, Auckland, New Zealand; 6 Department of Pneumology, University Hospital and University of Bern, Bern, Switzerland; Maastricht University, NETHERLANDS

## Abstract

**Background:**

Hypobaric hypoxia has been reported to cause endothelial cell and platelet dysfunction implicated in the formation of microvascular lesions, and in its extremes may contribute to vascular leakage in high altitude pulmonary edema or blood brain barrier disruption leading to cerebral micro-hemorrhage (MH). Platelet function in the development of microvascular lesions remained ill defined, and is still incompletely understood. In this study platelet- and endothelial cell-derived extracellular vesicles (PEV and EEV, respectively) and cell adhesion molecules were characterized in plasma samples of members of a high altitude expedition to delineate the contribution of platelets and endothelial cells to hypobaric hypoxia-induced vascular dysfunction.

**Methods and findings:**

In this observational study, platelet and endothelial cell-derived extracellular vesicles were analysed by flow-cytometry in plasma samples from 39 mountaineers participating in a medical research climbing expedition to Himlung Himal, Nepal, 7,050m asl. Megakaryocyte/platelet-derived AnnexinV^pos^, PECAM-1 (CD31) and glycoprotein-1b (GP1b, CD42b) positive extracellular vesicles (PEV) constituted the predominant fraction of EV in plasma samples up to 6,050m asl. Exposure to an altitude of 7,050m led to a marked decline of CD31^pos^ CD42^neg^ EEV as well as of CD31^pos^ CD42b^pos^ PEV at the same time giving rise to a quantitatively prevailing CD31^neg^ CD42b^low/neg^ subpopulation of AnnexinV^pos^ EV. An almost hundredfold increase in the numbers of this previously unrecognized population of CD31^neg^ CD42b^low/neg^ EV was observed in all participants reaching 7,050m asl.

**Conclusions:**

The emergence of CD31^neg^ CD42b^low/neg^ EV was observed in all participants and thus represents an early hypoxic marker at extreme altitude. Since CD31 and CD42b are required for platelet-endothelial cell interactions, these hypobaric hypoxia-dependent quantitative and phenotypic changes of AnnexinV^pos^ EV subpopulations may serve as early and sensitive indicators of compromised vascular homeostasis.

## Introduction

Humans who permanently live or work at, as well as mountaineers climbing to, high altitudes are at risk of developing high-altitude diseases, such as chronic or acute mountain sickness (AMS), high-altitude pulmonary edema (HAPE) and high-altitude cerebral edema (HACE) [1, 2].

Although the pathophysiology of high altitude diseases is only incompletely understood, endothelial damage is a hallmark in each of the different diseases. For example in HAPE, fluid transudation is aggravated due to endothelial damage resulting in vascular leakage. In HACE, disruption of the blood-brain barrier is leading to edema formation and the characteristic micro-hemorrhages (MH). Magnetic resonance imaging (MRI) in a recent prospective cohort study revealed new cerebral MH in 20%, that is, 3 of 15 climbers reaching an altitude of 7,050m [3]. The detection of MH and retinal hemorrhage in phenotypically healthy mountaineers at extreme altitude [3, 4] implicates an underestimated prevalence of substantial vascular dysfunction in “healthy” subjects exposed to hypoxia, which is yet prone to exacerbate anytime upon progression to severe hypoxic exposure.

Hypobaric hypoxia is the underlying cause of high altitude sickness by leading to sustained endothelial cell damage and platelet activation. Hallmarks of endothelial dysfunction include the expression of adhesion molecules and increased extracellular vesicle (EV) release into the circulation [5, 6]. Platelets are important for prevention of endothelial leakage, as they stop bleeding by forming clots at the site of endothelium injury [7]. Through this direct interaction with endothelial cells, platelets play an important role in the development of microvascular pathology. The interaction between platelets and the vessel wall is mediated by cellular receptors on the surface of platelets and endothelial cells, such as integrins and selectins [7]. Conditions caused by deficiency or dysfunction of CD42b, such as the Bernard-Soulier syndrome (BSS) [8], underline the functional relevance of these surface molecules. BSS is a hereditary bleeding disorder affecting the megakaryocyte/platelet lineage, indicating that CD42b is functionally required for normal primary hemostasis. Endothelial extracellular vesicles (EEV) and platelet extracellular vesicles (PEV) are released by activated and apoptotic endothelial cells and megakaryocytes/platelets, respectively, and carry surface molecules originating from their parental cells [9]. EEV and PEV carry an array of signaling molecules including cytokines, adhesion molecules, surface receptors, and bioactive lipids [10]. Megakaryocyte/platelet-derived PEV carry procoagulant factors, and trigger the binding of platelets to the subendothelial matrix [11]. Thus, PEV have been implicated to contribute to coagulation and vascular homeostasis, and endothelial dysfunction.

To assess the impact of extreme altitude on plasma EV of the 39 mountaineers of the medical research expedition to Mount Himlung Himal 2013, we analysed by flow cytometry plasma concentrations of platelet- and endothelial cell-derived extracellular vesicles (PEV and EEV) and quantitatively measured cell surface expression of AnnexinV, CD31, and CD42b. We report here the emergence of a previously unrecognized CD31^neg^ subpopulation population of AnnexinV^pos^ EV of all mountaineers reaching 7,050m asl that may serve as an indicator of the initiation of vascular dysfunction.

The challenging logistics of such a high altitude medical expedition precluded a large collection of plasma samples limiting the number of parameters that could be comprehensively investigated. Thus, the observations reported will benefit from future detailed systematic in vitro analyses. Especially more in depth characterization of the emerging type of CD31^neg^ CD42^low/neg^ AnnexinV^pos^ EV will reveal the cellular origin, molecular composition, and function of this previously unrecognized population of EV.

## Methods and material

### Experimental setting

HiReach 2013 was a high altitude medical research field study to Mount Himlung Himal (7126 m), Nepal. 39 healthy and physically fit subjects were examined by baseline (PR) and post-expedition (PO) testings at 550 m asl, eight to nine weeks before and four to five weeks after climbing to Himlung Himal. The study was approved by the relevant institutional ethical committee, (the Kantonale Ethik Komitee, KEK 226/12) and was registered on clinicaltrials.gov (NCT01953198). Informed consent was obtained from all subjects before study inclusion and all data were anonymized for analysis. During the climb examinations were performed one day after arrival each at Base Camp (BC1) at 4844m, in Camp 2 (C2) at 6022 m, and in Camp 3 (C3) at 7050 m. Subject recruitment procedures and ascent protocol has been published previously [3].

### Blood sample collection

Citrate-plasma samples from climbers were prepared by centrifugation of peripheral blood samples for 10 minutes at 2,000 g (EBA 20, Hettich AG, Bäch, Switzerland) afterwards 300μl of plasma aliquots were frozen at -40°C to -60°C on-site and stored at -80°C until analysis.

### Flow cytometry of extracellular vesicles in plasma

**Fig 1:**
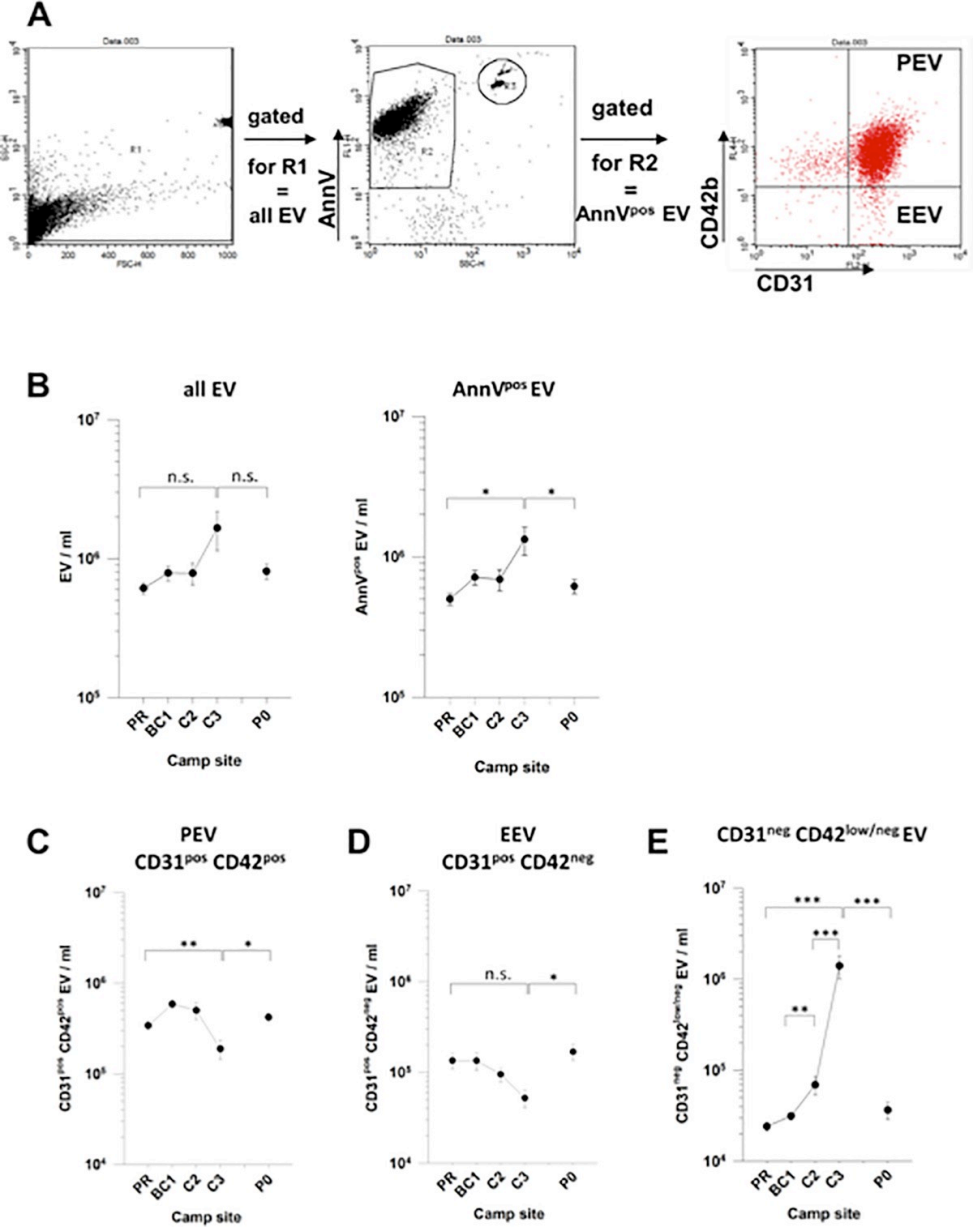
Extracellular vesicle contents in the plasma of mountaineers over the course of the high altitude expedition. Defined extracellular vesicle types were quantified by flow cytometry in plasma samples drawn from mountaineers at the indicated camp sites. (A) Gating strategy for identification of PEV and EEV among AnnexinV^pos^ extracellular vesicles by expression of CD31 and CD42b. (B) Plasma contents of total EV (left panel) and AnnexinV^pos^ EV (right panel). (C-E) Plasma contents of (C) CD31^pos^ CD42b^pos^ PEV, (D) CD31^pos^ CD42b^neg^ EEV, and (E) CD31^neg^ CD42b^low/neg^ EV emerging at high altitude beyond 6.000 m asl. Shown are means +/- SE of data from all individuals reaching the indicated camp sites. Statistical significance is indicated by “*”, “**”, or “***” representing p-values of p<0.05, p<0.01, or p<0.001, respectively; “n.s.” = not significant.

Effects of hypobaric hypoxia at high altitude on extracellular vesicles (EV) in plasma were analyzed by flow cytometry (Fig 1A). This is currently the most widely used and accepted methodology for EV analyses [12]. In recent years, EV attracted much academic as well as clinical attention due to their assumed function as signaling units and biomarkers in a plethora of physiological and pathophysiological contexts [13, 14]. Large consortia strive to assess and establish protocols for standardization and quality control of qualitative as well as quantitative characterization of EV [12–14] as prerequisites for reliable routine application. As previously described (17) we here followed basic, and well-established protocols for flow cytometrical analysis of EV, [15–17]. EV were identified by size and granularity, and further specified by phosphatidylserine (PS) surface exposure (AnnexinV-binding). Platelet-derived (P)EV and endothelial cell-derived (E)EV were defined by platelet endothelial cell adhesion molecule (PECAM-1, CD31) surface antigen expression. The EV origin was further differentiated by platelet glycoprotein Ib alpha chain (GPIb, CD42b) expression on PEV (CD42b-positive) versus EEV (CD42b-negative). Flow cytometry of surface expression of CD31 (PECAM-1) and CD42b (GPIb, CD42b) on EV is a widely used method to discriminate PEV (CD31^pos^ CD42b^pos^) from EEV (CD31^pos^ CD42b^neg^)[15]. Plasma EV were analyzed as published previously [16]. Briefly, plasma samples were thawed at 37^o^ C and centrifuged at 17,000 g for 10 min at RT. Pelleted material was resuspended in AnnV-binding buffer according to manufacturer´s instructions and aliquoted into BD Truecount Tubes (all BD Biosciences, Heidelberg, Germany). Samples were incubated with APC anti human CD42b and PE anti human CD31 in the dark at RT for 15 min before analysis with a FacsCalibur (all BD Biosciences). For analysis, gating started with total plasma EV, including the Truecount microbeads for quantification of EV/ml (Fig 1A, left), before AnnV^pos^ EV (Fig 1A, middle) were further analyzed for CD31 and CD42 expression in order to differentiate between CD31^pos^ CD42b^pos^ PEV and CD31^pos^ CD42b^neg^ EEV (Fig 1A, right).

### Measurement of soluble cell adhesion molecules

Plasma levels of human Vascular Endothelium Cadherin (VE-Cadherin), Intercellular Adhesion Molecule 1 (ICAM-1), and Vascular Cell Adhesion Molecule 1 (VCAM-1) were determined by specific ELISA (DuoSet ELISA: #DY809 (VCAM-1), #DY720 (ICAM-1), #DY938-05 (VE-Cadherin); R&D Systems, Inc., USA) according to the manufacturer’s instructions. Plasma samples were centrifuged at 17,000 g for 10 minutes and supernatant was diluted 1:100 for detection of VE-Cadherin, 1:250 for ICAM-1 and 1:500 for VCAM-1 in 1% BSA/PBS. All samples were measured in duplicates. Absorbance was determined at 450 nm with a multimode microplate reader (EnSpire, Perkin Elmer, USA) and plasma concentrations were calculated by four-parameter nonlinear regression model (4-PL) curve-fitting.

### Statistics

According to the Shapiro-Wilk normality test, the data were largely not normally distributed. Therefore, p-values were determined by Mann-Whitney Rank Sum Test.

## Results

Thirty nine subjects participated the expedition of whom 35 reached Camp 2 and 15 Camp 3. Details on time course of the expedition and subjects characteristics are given in Table 1. Additional details have been published previously [3, 18]. Most importantly and as expected, arterial oxygen saturation correlated inversely proportional with altitude (Table 1). Despite increasingly high variation at extreme altitudes, arterial oxygen saturation differed highly significantly (p < 0.001) for any comparison of camp sites except for the comparison between PR and P0.

**Table 1:** Details of the time course of the expedition, study sites altitude, and subjects examined.

Camp site					
	PR^¶^	BC1	C2	C3	PO
**Course of the expedition**					
**Time since Kathmandu**	8/9 weeks before	5 days	11 days	23/24 days	4/5 weeks after expedition
**Altitude**	550 m	4800 m	6050 m	7050 m	550 m
**Subject characteristics**					
Mean age of 45.5 ± 12.1 years					
**Subjects [n]**	39	39	36	15	39
**SaO**_**2**_ **[%]**	97.6 ± 0.8	83.8 ± 4.6	72.7 ± 8.8	68.8 ± 9.6	97.4 ± 0.7

^¶^Abbreviations: PR = pretest examination, BC1 = Base Camp, first assessment, C2 = Camp 2, C3 = Camp 3, PO = post expedition examination

### Altitude dependent decline of CD31 expressing PEV and EEV associated with the emergence of a CD31^neg^ subpopulation of AnnexinV^pos^ EV

Plasma concentrations of all EV increased with increasing altitude from ~6x10^5^ EV/ml at PR to about 1.7x10^6^ EV/ml at C3 and declined to baseline levels within 4 to 5 weeks after returning to PO at 550m asl (Fig 1B). Similar kinetics were observed for AnnexinV^pos^ EV that constitute the majority of all EV (Fig 1B). Analysis of subpopulations of AnnexinV^pos^ EV revealed that CD31^pos^/CD42^pos^ PEV constituted the vast majority of AnnexinV^pos^ EV at altitudes up to 6,022m (Fig 1C). It is worth emphasizing that CD31^pos^/CD42b^pos^ EV are composed of both platelet- and megakaryocyte-derived EV [19]. Thus, the term PEV reflects the entire megakaryocyte/platelet lineage as cellular origin. Compared to baseline levels (PR), PEV slightly increased at 4,844m asl, remained stable at 6,022m, yet markedly dropped at 7,050m asl (C3; Fig 1C). In contrast, the number of CD31^pos^/CD42^neg^ EEV remained constant from PR to 4,844m (BC1) and then gradually dropped from 6,022m to 7,050m to about 5x10^4^ EEV/ml (Fig 1D). Surprisingly, small numbers of a previously unrecognized population of CD31^neg^ EV were detectable at PR, which gradually increased to become at C3 the quantitatively dominant population of AnnexinV^pos^ EV (Fig 1E).

**Fig 2:**
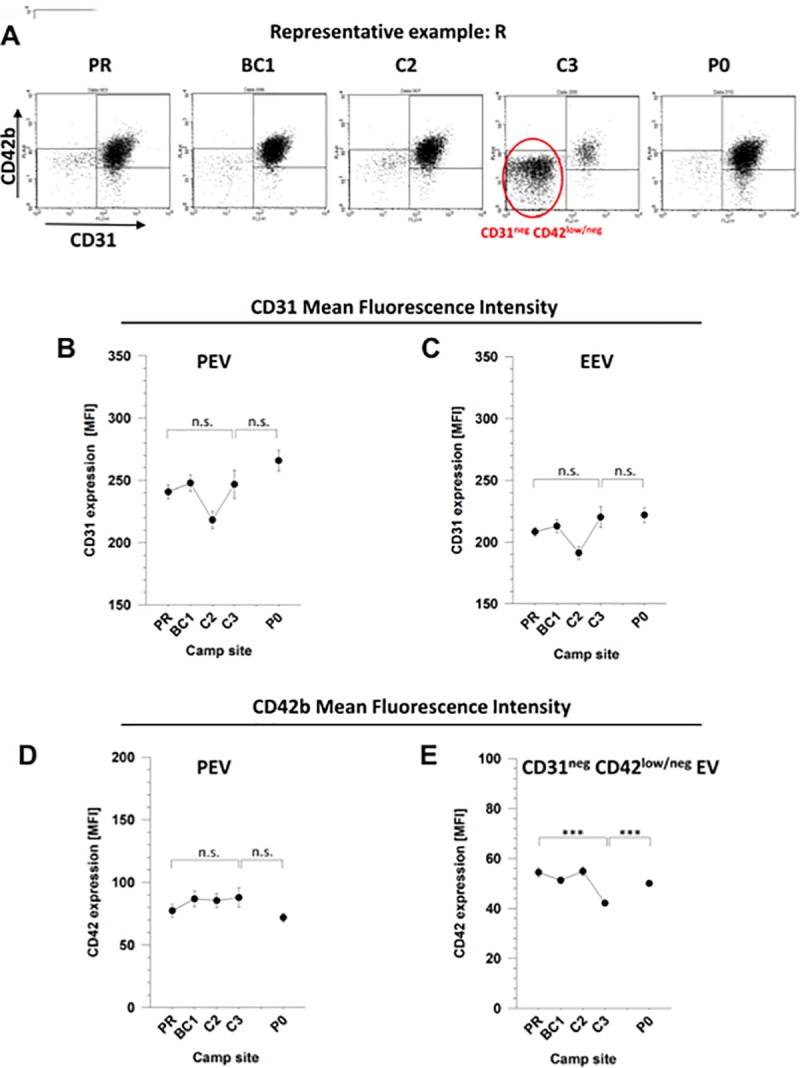
Expression levels of CD31 and CD42b on AnnexinV^pos^ extracellular vesicles. Plasma samples of mountaineers were analysed by flow cytometry as specified in Fig 1. Expression levels of the markers CD31 and CD42b were determined as mean fluorescence intensity (MFI). (A) Dot plots of expression levels of CD31 and CD42b on AnnV^pos^ EV of a representative individual (“R”) are shown at each altitude. The emergence of a large amount of CD31^neg^ CD42b^low/neg^ EV at C3 is highlighted. (B-C) Expression levels of CD31 on PEV (B) and EEV (C). (D-E) Expression levels of CD42b on PEV (D) and CD31^neg^ CD42b^low/neg^ EV (E). Shown are means +/- SE of data from all individuals reaching the indicated camp sites. Statistical significance of p<0.001 is indicated by “***”; “n.s.” = not significant.

The discrepancy between the increase of EV numbers and the decline of CD31^pos^ EV prompted us to analyse CD31 expression on PEV and EEV in greater detail. Flow cytometry analysis of samples from a representative individual (“R”) indicate a stable distribution of PEV and EEV up to C2 (6,022m asl). At C3 (7,050m) a large population of CD31^neg^ EV emerged (Fig 2A). At post-expedition analysis, this CD31^neg^ EV population declined back to normal levels. At C3, the mean fluorescence intensities of CD31 on PEV and EEV was comparable to those observed at 550m and 4,800m (Fig 2B and 2C). Similar results were obtained for CD42b on PEV (Fig 2D). These data suggest that the remaining small populations of CD31^pos^ PEV and EEV express CD31 or CD42b (PEV) at normal densities. In contrast, the emerging CD31^neg^ EV showed a continuum of low to absent CD42 expression, i.e. slightly above and below the threshold for background staining (Fig 2E).

**Fig 3:**
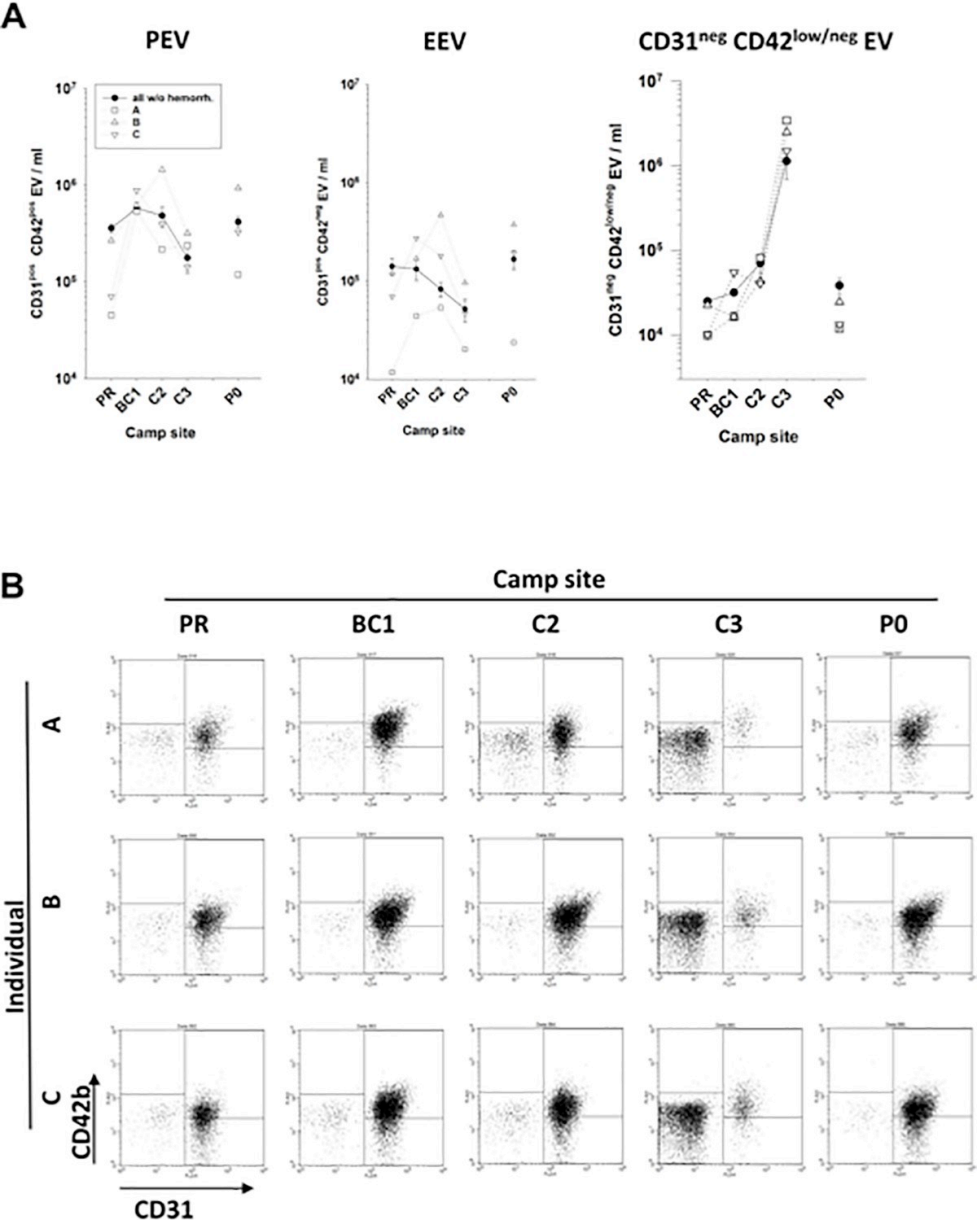
Analysis of the AnnexinV^pos^ extracellular vesicles of individuals with cerebral microhemorrhages. Plasma samples of mountaineers were analysed by flow cytometry as described in Fig 1. (A) Amounts of PEV, EEV, and CD31^neg^ CD42b^low/neg^ EV in the plasma of the individuals A, B, and C (open symbols) in comparison to the mean +/- SE values of all non-hemorrhagic peers (closed symbols). (B) Dot plots of CD31 and CD42b expression on AnnV^pos^ EV of individuals A, B, and C at the various camp sites.

The patterns of PEV and EEV kinetics of the three individuals suffering from hypobaric hypoxia-induced microhemorrhages in the brain (climbers A, B, and C as described by Kottke et al. [3]) were of special interest. However, the individual CD31 and CD42b expression profiles of PEV and EEV of these climbers were mostly inconspicuous (Fig 3).

**Fig 4:**
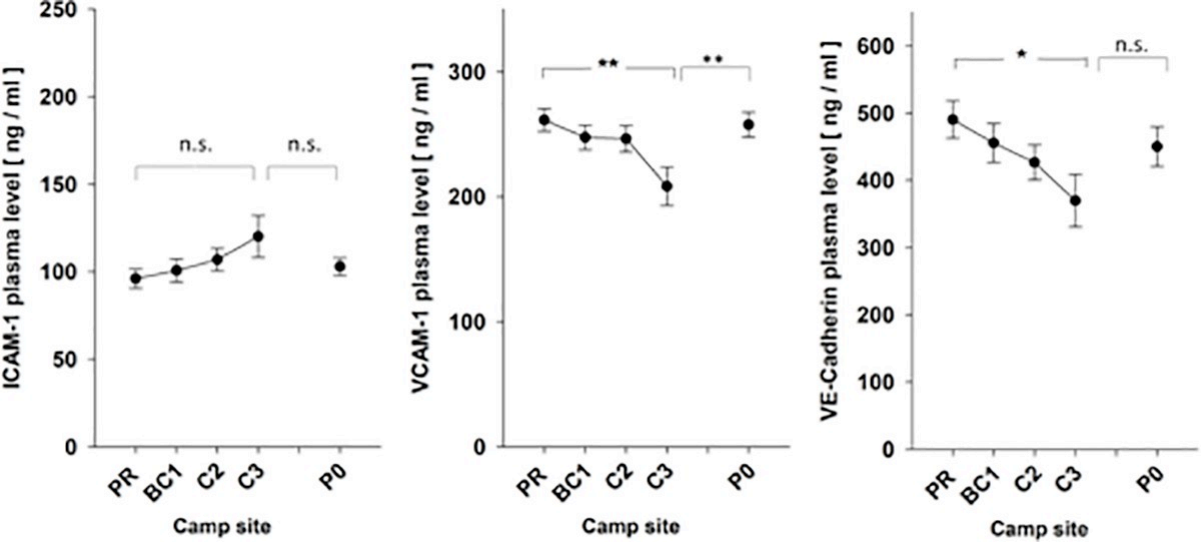
Levels of soluble ICAM-1, VCAM-1, and VE-Cadherin in plasma over the course of the high altitude expedition. Plasma contents of soluble ICAM-1, VCAM-1, and VE-Cadherin were quantified by specific ELISA. Shown are the means +/- SE for all individuals reaching the indicated camp sites. Statistical significance is indicated by “*” or “**” representing p-values of p<0.05 or p<0.01, respectively; “n.s.” = not significant.

The quantitative and phenotypic changes of AnnexinV^pos^ EV suggested a perturbed homeostasis of endothelial cells and/or platelets. To assess further markers of endothelial cell and/or platelet dysfunction, we analyzed as proxy the plasma concentrations of soluble cell adhesion molecules ICAM-1, VE-Cadherin and VCAM-1. As shown in Fig 4, ICAM-1 slightly but not significantly increased with altitude. In contrast, the concentrations of VCAM-1 and VE-cadherin significantly dropped at C3, that is, by 20%, p_(PR vs. C3)_ = 0.003, and 16%, p_(PR vs C3)_ = 0.02, respectively, indicating a cellular response of endothelial cells to hypobaric hypoxia at 7,050m.

Notably, only 15 out of 39 participants dropped reached camp 3. To rule out a possible bias, we plotted selectively the data from only those climbers that reached C3 (EVs and soluble adherence molecules). As shown in supplemental S1 Fig and S2 Fig, the overall picture remained the same, that is, the altitude-dependet changes of EEV, PEV, and AnnexinVpos, CD31neg, CD42blow/neg EV populations of C3 climbers were comparable to those observed with entire population.

## Discussion

Accumulating evidence suggests that endothelial cell dysfunction is a primary factor of hypobaric hypoxia-induced high altitudes sickness [2]. According to the prevailing opinion, endothelial cells are both primary targets of hypobaric hypoxia as well as important determinants of the development of vascular damage causing AMS, HACE, or HAPE. Since EEV serve as marker of endothelial activation or apoptosis [9], we here phenotyped EEV in plasma samples from mountaineers during a high altitude medical expedition. Surprisingly, the major observation revealed by this field study is a previously unrecognized population that sharply emerged at 7,050m to become the quantitatively predominating EV population in all participants and thus can be regarded as a new and early marker of hypobaric hypoxia-induced perturbance of vascular homeostasis. The cellular origin of this new class of CD31^neg^ CD42b^low/neg^ EV has not been resolved. This was due mainly to logistic limitations of such a medical expedition, particularly the small plasma sample volume per person and camp site and the large time intervals between sample takings. Notwithstanding, the finding that EEV and PEV concomitantly and significantly declined at 7,050m strongly suggests that the emerging population of AnnexinV^pos^ CD31^neg^ CD42b^low/neg^ EV originates from both activated/apoptotic endothelial cells and megakaryocytes/platelets. However, the possibility remains that hypobaric hypoxia induces apoptosis of third party cell types and subsequent release of AnnexinV^pos^ CD31^neg^ EV. Future studies using purified platelets/megakaryocytes and endothelial cells cultured in hypobaric hypoxic chambers will corroborate the cellular origin and define the molecular mechanisms underlying the emergence of the AnnexinV^pos^ CD31^neg^ CD42b^low/neg^ EV population. Specifically, it should be worthwhile to explore whether this population is generated by activation of sheddases or secondary to down-regulation of the corresponding genes, which will provide further insights into the hypobaric hypoxic stress response of megakaryocytes/platelets and endothelial cells, respectively. The generation of AnnexinV^pos^ CD31^neg^ CD42b^low/neg^ EV population under in vitro conditions, would make this EV population amenable to functional investigations aiming at their possible impact on bleeding disorders or vascular leakage.

A previous study of participants of a climbing expedition to Peak Lenin revealed that the fraction of CD31^pos^ EEV did not change significantly with altitude up to 6,210m asl [5]. This is consistent with the findings of the present study, where the numbers of CD31 expressing EEV and PEV remained rather constant up to 6,022m asl. As shown in Fig 1E, the decline of CD31 expressing PEV and EEV became obvious only with climbers reaching an altitude of 7,050m asl.

CD31 is fundamental for prevention of endothelial apoptosis and for the maintenance of vascular integrity [20–23], Loss of CD31 surface expression by endothelial cells is secondary to proteolytic CD31 shedding caused by metalloproteinases [24]. The decline of CD31^pos^ EEV and emergence of CD31^neg^ EV might well reflect loss of CD31 expression by endothelial cells at 7,050m. Thus our observations suggest but do not prove that apoptotic endothelial cells generate part of the CD31^neg^ EV population at 7,050m.

Next to CD31, CD42b can be shed from the platelet surface by metalloproteinases [25], e.g. by the TNFalpha converting enzyme (TACE), which is activated via p38 MAPK and endogenously produced reactive oxygen species (ROS) [26]. During sojourns at extreme altitude, chronic hypoxia leads to the production of superoxide and other ROS, because reduced mitochondrial respiration is associated with leakage of activated oxygen from mitochondria during oxidative phosphorylation [27, 28]. Platelets contain small numbers of fully functional mitochondria that can generate significant concentrations of ROS [29]. Thus, it seems reasonable to assume that increasing levels of ROS activate metalloproteinases at 7,050m asl leading to phenotypic and functional changes of platelets thereby tipping the balance between altitude-related adaptive processes and vascular disease.

CD42b has been shown to be functionally required for primary hemostasis. Shedding of CD42b leads to a dramatic attenuation of platelet function [30]. A deficiency or dysfunction of CD42b is the underlying defect of the Bernard-Soulier syndrome (BSS), a hereditary bleeding disorder affecting the megakaryocyte/platelet lineage and characterized by bleeding tendency [8]. Indeed, bleeding is a frequent disorder among climbers at high altitude. In particular, retinal bleeding has been reported by us and other investigators [4, 31] affecting as many as 93% of climbers directly after returning from 6,865m asl. Interestingly, during a previous high altitude medical research expedition to Muztagh Ata (7,549m asl) we previoulsy detected at extreme altitude a significant decrease of von Willebrand factor (vWF), a ligand of CD42b [32]. This was associated with procoagulant changes including a prolonged PT and aPTT, increased D-dimers and Activated Protein C (APC)-resistance. These changes of parameters are characteristic of vWF consumption during ascent consistent with activation or disruption of the endothelial structure. The results of this study suggest that vascular dysfunction at extreme altitude is not only secondary to hypoxia-induced direct endothelial damage but also promoted indirectly, i.e. by compromised interactions between endothelial cells and functionally defective platelets. It will be interesting to assess a possible functional relevance of the emerging CD31^neg^ CD42^low/neg^ EV in platelet -endothelial cell interactions. Given that EV are well-established carriers of micro RNAs, our previously reported findings of increased miRNA-190 and miRNA-17 concentrations at C3 (18) suggest a role of this quantitatively dominant CD31^neg^ CD42^low/neg^ EV population in hypoxia-induced vascular remodeling.

Soluble cell adhesion molecules were assessed to further characterize endothelial dysfunction under hypoxic conditions. VCAM-1 is an adhesion molecule that is upregulated upon endothelial activation [33, 34] and mediates endothelial leukocyte adherence and trafficking, therefore promoting inflammatory immune responses. The soluble forms are released into circulation by proteolytic cleavage. Circulating VCAM-1 has been shown to be a promising marker of endothelial dysfunction [35, 36]. VE-cadherin is a transmembrane protein exclusively expressed by endothelial cells [37], whose extracellular domain forms dimers with VE-cadherin on adjacent cells [38]. VE-cadherin-induced activation of phosphoinositide 3-kinase (PI3K) and Akt kinase, results in up-regulation of the tight-junction adhesive protein claudin 5, thus promoting junction tightening [39]. VE-cadherin activates and maintains endothelial cell quiescence by promoting expression of genes implicated in inhibition of cell proliferation and apoptosis [40] and by limiting growth factor receptor signaling [41]. ICAM-1 is constitutively expressed on vascular endothelial cells as well as some lymphocytes and monocytes [42], but can be induced by inflammatory cytokines such as interleukin-1, interferon gamma[43], and TNF alpha[44]. ICAM-1 participates in leukocyte-epithelial cell interactions and enables leukocyte migration from the capillary bed into tissue[45].

We hypothesized that altitude-related endothelial dysfunction would lead to progressive shedding of ICAM-1, VCAM-1 and VE-cadherin into plasma. Indeed, we detected a slight yet insignificant altitude-dependent increase of ICAM-1. In contrast, VCAM-1 and VE-cadherin significantly declined at 7,050m. This surprising observation merits some consideration. The plasma concentration of adhesion molecules is the result of many processes comprising in general a) production (e.g. cell surface expression, shedding) and b) plasma half-life time depending on e.g. binding to cognate ligands, internalization by cell-bound ligands, or proteolytic degradation. Thus, the changes of plasma concentrations of ICAM-1, VCAM-1 and VE-cadherin do not necessarily correlate with the phenotype of EV released by endothelial cells or platelets under hypobaric hypoxic conditions.

Although the functional implications of our study remain unresolved, the emerging CD31^neg^ CD42b^low/neg^ subpopulation of AnnexinV^pos^ EV is a new and potentially valuable biomarker for hypobaric hypoxic stress.

## Supporting information

S1 Fig Extracellular vesicle contents in the plasma of those mountaineers reaching camp site C3 over the course of the high altitude expedition. Defined extracellular vesicle types were quantified by flow cytometry in plasma samples drawn from mountaineers at the indicated camp sites. Gating strategy shown in Fig 1A. (A) Plasma contents of total EV (left panel) and AnnexinV^pos^ EV (right panel). (B-D) Plasma contents of (B) CD31^pos^ CD42b^pos^ PEV, (C) CD31^pos^ CD42b^neg^ EEV, and (D) CD31^neg^ CD42b^low/neg^ EV emerging at high altitude beyond 6.000 m asl. Shown are means +/- SE of data from those individuals reaching camp site C3. Statistical significance is indicated by “*”, “**”, or “***” representing p-values of p<0.05, p<0.01, or p<0.001, respectively; “n.s.” = not significant.
(TIF)

S2 Fig Levels of soluble ICAM-1, VCAM-1, and VE-Cadherin over the course of the high altitude expedition in plasma of those individuals reaching C3. Plasma contents of soluble ICAM-1, VCAM-1, and VE-Cadherin were quantified by specific ELISA. Shown are the means +/- SE for those 15 individuals reaching camp site C3. Statistical significance is indicated by “*” representing a p-value of p<0.05; “n.s.” = not significant.
(TIF)

S1 Table Dataset for all EV in Fig 1B. (XLSX)

S2 Table Dataset for AnnV^pos^ EV in Fig 1B. (XLSX)

S3 Table Dataset for PEV in Fig 1C. (XLSX)

S4 Table Dataset for EEV in Fig 1D. (XLSX)

S5 Table Dataset for CD31^neg^ CD42^low/neg^ EV in Fig 1E. (XLSX)

S6 Table Dataset for PEV in Fig 2B. (XLSX)

S7 Table Dataset for EEV in Fig 2C. (XLSX)

S8 Table Dataset for PEV in Fig 2D. (XLSX)

S9 Table Dataset for CD31^neg^ CD42^low/neg^ EV in Fig 2E. (XLSX)

S10 Table Dataset for ICAM-1 in Fig 4. (XLSX)

S11 Table Dataset for VCAM-1 in Fig 4. (XLSX)

S12 Table Dataset for VE-Cadherin in Fig 4. (XLSX)
