# Supplementary figures and images for: Emergence of AnnexinVpos CD31neg CD42blow/neg extracellular vesicles in plasma of humans at extreme altitude
Source: PLoS One. 2019 Aug 1;14(8):e0220133. doi: 10.1371/journal.pone.0220133 (PMC6675110; doi:10.1371/journal.pone.0220133)

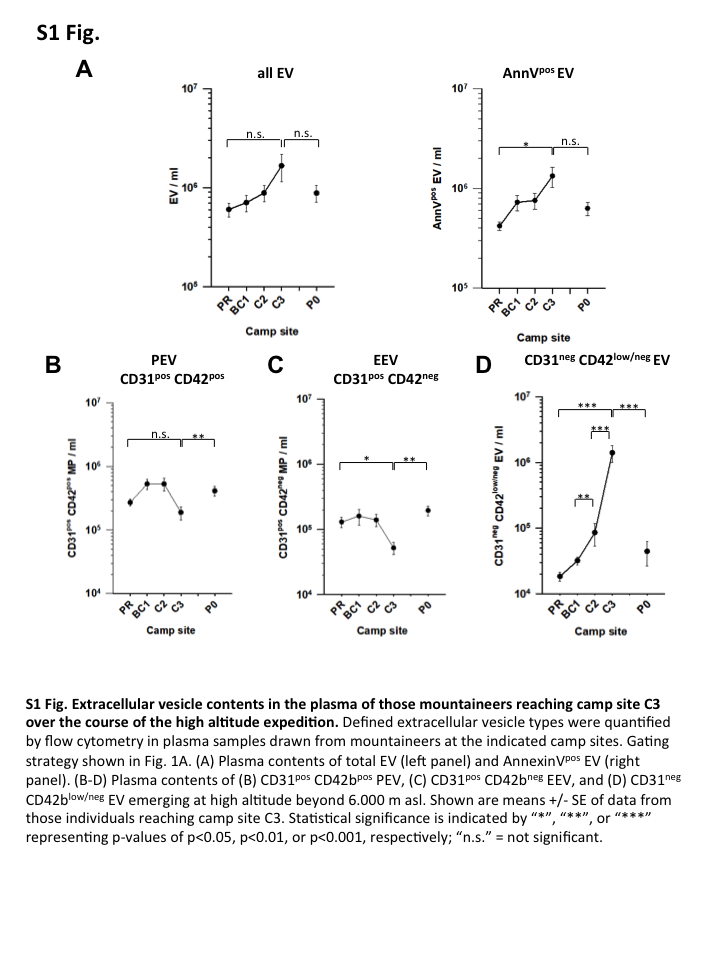

Supplement: S1 Fig — Defined extracellular vesicle types were quantified by flow cytometry in plasma samples drawn from mountaineers at the indicated camp sites. Gating strategy shown in Fig 1A. (A) Plasma contents of total EV (left panel) and AnnexinVpos EV (right panel). (B-D) Plasma contents of (B) CD31pos CD42bpos PEV, (C) CD31pos CD42bneg EEV, and (D) CD31neg CD42blow/neg EV emerging at high altitude beyond 6.000 m asl. Shown are means +/- SE of data from those individuals reaching camp site C3. Statistical significance is indicated by “*”, “**”, or “***” representing p-values of p<0.05, p<0.01, or p<0.001, respectively; “n.s.” = not significant. (TIF) [file pone.0220133.s001.tif]

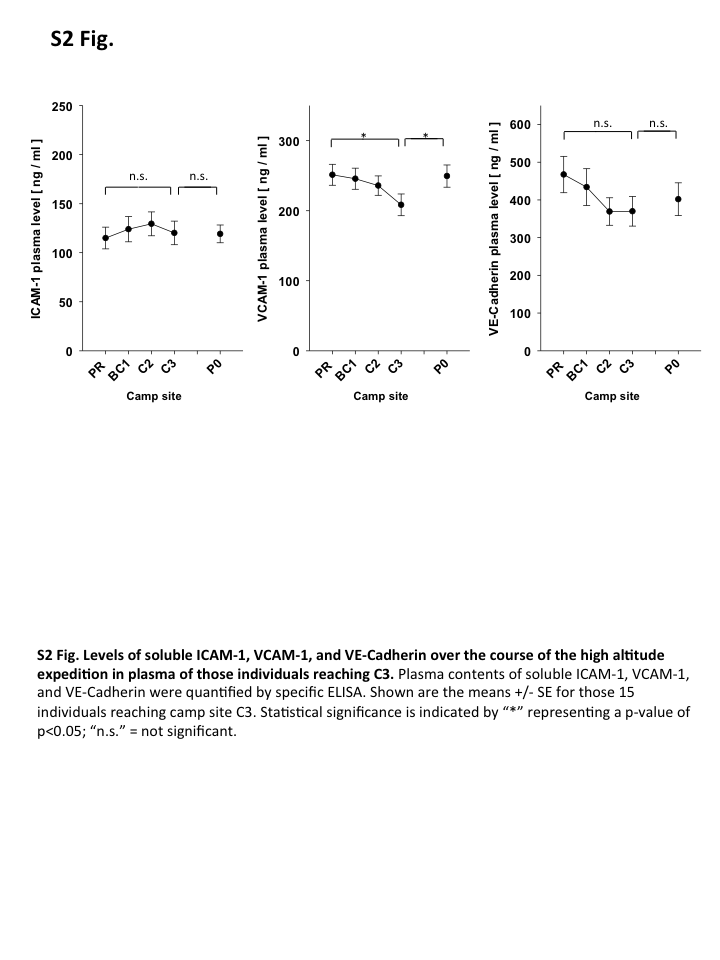

Supplement: S2 Fig — Plasma contents of soluble ICAM-1, VCAM-1, and VE-Cadherin were quantified by specific ELISA. Shown are the means +/- SE for those 15 individuals reaching camp site C3. Statistical significance is indicated by “*” representing a p-value of p<0.05; “n.s.” = not significant. (TIF) [file pone.0220133.s002.tif]
